# Scoping the Potential for a Digital Led Recovery from COVID-19 in Africa

**DOI:** 10.1007/s44232-022-00007-7

**Published:** 2022-10-06

**Authors:** Rashmi Banga, Karishma Banga

**Affiliations:** 1 Division on Globalization and Development Strategies, UNCTAD, Geneva, Switzerland; 2 Institute of Development Studies (IDS), University of Sussex, Brighton, UK

**Keywords:** Covid-19, Digital economy, Digital led recovery, Digital divide, Electronic transmissions, Digitally delivered services

## Abstract

In this paper, we analyse the implications of the COVID-19 pandemic for Africa's trade in digitizable products, digitally deliverable services (DDSs), and e-commerce, and we outline the challenges of a digitally led recovery from the pandemic for African countries. The analysis is based on macro- and micro-level data and a review of extant literature. We find that almost 60% of the global exports of digital products in 2020 originated from the EU, with another 16% from North America. Africa's share in global exports of digital products, by contrast, was less than 1%. In the case of intra-Africa trade, exports of digital products are concentrated in just a few countries. Online supply in services is found to be more resilient to negative shocks from the pandemic compared to other modes of supply. We also find that e-commerce has important potential for economic recovery, but that its impact in Africa is constrained by high internet costs, weaknesses in postal services and capacities, cross-border trade costs, and lags in electronic infrastructure and digital payment systems. Based on the results of the study, we propose policies at the national, regional, and international levels to leverage digital trade for post-COVID recovery in Africa.

## Introduction

The COVID-19 pandemic has underscored the importance of strengthening digital trade in Africa. The spread of the pandemic led to a contraction in the global economy of 3.9% in 2020. By comparison, Africa’s GDP contracted by 3.8% (UNCTAD, 2021a, 2021b). In Sub-Saharan Africa, output contracted by an estimated 3.7% in 2020, with a decline in per capita income of 6.1% (Global Economic Prospects, 2021). This economic slowdown, accompanied by periodic lockdowns in most countries, has adversely affected international trade. Accordingly, global merchandise trade contracted by 5.3% in 2020, while Africa’s merchandise exports and imports declined by 8.1 and 8.8%, respectively (WTO, 2021). The decline in the mobility of people also led to a drastic decline in global trade in services, which fell by 15% in 2020. The worst hit service sectors were travel and tourism, which comprise major service exports of Africa. The number of international tourist arrivals between April and June 2020 was 98% lower than in the same period in 2019 (UNWTO, 2021). The International Air Transportation Association estimates that Africa’s aviation industry lost $2 billion in 2020 (IATA, 2020). Further, demand for air travel is not expected to reach its pre-COVID-19 levels before 2023.

However, amidst this global slowdown and contraction in international trade in goods and services, digital trade experienced unprecedented growth, bringing huge gains to digital exporters. Digital trade covers digitally ordered but physically delivered products (i.e., e-commerce, such as importing a book from Amazon); and digitally ordered and digitally delivered products and services (i.e., importing digitalized products such as e-books and importing services via Mode 1 (services provided by phone, fax, or electronic means), e.g., financial services). During the pandemic, global e-commerce grew by 27.6%. But the Middle East and Africa witnessed relatively little growth, at 19.8% (OBERLO, 2020). Further, Africa’s share in digital exports of goods and services is negligible. While other regions like Asia and the Pacific have regained lost export revenues through digital exports during the pandemic, African countries are losing out on this opportunity. The growing digital divide between Africa and the rest of the world, as well as within Africa, may eclipse the growing opportunities offered by global digital trade. Therefore, it is important for African countries to implement policies at the national, regional, and international levels to boost African digital trade.

In this context, this paper reviews and analyses the implications, prospects, and challenges of the COVID-19 pandemic for Africa's trade in digitizable products, digitally deliverable services (DDSs), and e-commerce. We examine digital trade in African countries and the extent of the digital divide between Africa and the rest of the world, as well as between African countries. We assess the implications of the COVID-19 pandemic for Africa’s digital divide and for its digital trade in goods and services, with a particular focus on Africa’s e-commerce trade. To strengthen Africa’s digital trading capacity, we recommend policies at the national, regional, and continental levels, including policies needed at the international level, i.e., at the WTO.

The paper is organized as follows: Sect. 2 examines the existing global digital divide using information and communication technologies (ICT) development indicators. Section 3 discusses the demand- and supply-side effects of the COVID-19 pandemic on digital infrastructure in Africa. Section 4 maps Africa’s trade in digital products, digital services, and e-commerce by examining Africa’s digital trade with the world and intra-African digital trade. Section 5 provides the results of an analysis undertaken using the World Bank’s “Impact of COVID survey” database on how growth outcomes have differed across digital responses to COVID. Section 6 discusses the policies that are needed at the national level to strengthen digital trade in goods, services, and e-commerce in Africa. Section 7 proposes a digital cooperation agenda at the regional level. Section 8 discusses international cooperation for a digital transformation of Africa and an increase in digital trade. Section 9 concludes and discusses ways forward.

##  Growing Digital Divide: Africa

COVID-19 has exposed the existing global digital divide, especially for African countries. At the outset, internet penetration was much lower in Africa than in other regions (Table 1). In Europe and in America, approximately 82% and 76% of people have access to internet, respectively, but this falls to 29% in Africa, which is well below even the developing country average of 44%.

**Table 1:** Key ICT indicators for the ITU/BDT regions (totals and penetration rates)

		Africa	Arab states	Asia and Pacific	Europe	Americas	World	Developed countries	Developing countries
Fixed telephone subscriptions	Millions	7	37	386	226	216	915	439	476
	Per 100 inhabitants	0.7	8.7	9	32.9	21.3	11.9	34.3	7.4
Mobile cellular telephone subscriptions	Millions	836	424	4709	818	1139	8283	1684	6600
	Per 100 inhabitants	80.1	99	110.3	119.2	112.7	107.8	131.8	103
Active mobile broadband subscriptions	Millions	335	265	3220	669	1001	5702	1583	4119
	Per 100 inhabitants	32.1	62	75.4	97.4	99.1	74.2	123.9	64.3
Fixed broadband subscriptions	Millions	5	33	613	221	216	1134	424	710
	Per 100 inhabitants	0.5	7.7	14.3	32.1	21.4	14.8	33.2	11.1
Population covered by at least a 3G mobile network	Millions	788	388	4096	675	964	7128	1249	5879
	Per 100 inhabitants	75.6	90.7	95.9	98.4	95.4	92.8	97.7	91.8
Individuals using the internet	Millions	299	234	1901	568	774	3969	1108	2852
	Per 100 inhabitants	28.6	54.6	44.5	82.5	76.7	51.4	86.7	44.4

Source: ITU World Telecommunication/ICT Indicators database

Updated: November 2020

A 10% increase in mobile broadband penetration in Africa could increase the GDP per capita of the continent by 2.5% (ITU, 2019), but at present, active mobile-broadband subscriptions in Africa per 100 inhabitants are half those of developing countries and less than half the world average. The digital divide is specifically stark when we look at access to fixed broadband: 33% of people in developed countries have a fixed-broadband subscription, which falls to 11% of people in developing countries and less than 1% in the case of Africa. More than 90% of people have access to 3G networks across regions, but only 75% have access to 3G in the case of Africa (Table 1).

The digital divide is also visible within Africa. More than half of the population in some African countries have access to internet, such as Morocco, Tunisia, Seychelles, South Africa, and Gabon (Table 2). But in other African countries, such as Comoros, South Sudan, Chad, and Burundi, internet penetration, measured as the percentage of population with access to internet, is below 10%. Overall, African countries fare better in terms of accessing internet through mobile phones than fixed broadband penetration, which in several African countries is less than 1% (Table 2).

**Table 2:** Existing digital divide within Africa, 2019

Country	Internet penetration (%)	Fixed broadband subscriptions (per 100 people)	Mobile cellular subscriptions (per 100 people)
Morocco	64.80	4.80	127.95
Tunisia	64.19	10.20	126.31
Seychelles	58.77	27.60	198.15
South Africa	56.17	2.14	165.60
Gabon	50.32	1.03	137.75
Botswana	41.41	2.14	162.64
Ghana	37.88	0.19	134.32
Namibia	36.84	2.54	113.19
Sudan	30.87	0.08	77.11
Lesotho	29.79	0.30	74.49
Senegal	29.64	0.93	109.72
Uganda	23.71	0.07	57.37
Cameroon	23.20	1.55	82.70
Kenya	22.57	0.93	103.77
Rwanda	21.77	0.07	76.49
Mauritania	20.80	0.24	104.09
Mozambique	20.77	0.23	48.65
Tanzania	16.00	1.79	82.21
Angola	14.34	0.37	46.60
Malawi	13.78	0.06	47.78
Sierra Leone	13.24		86.13
Mali	12.72	0.73	116.62
Comoros	8.48	0.14	67.60
South Sudan	7.98	0.00	20.09
Chad	6.50	0.00	48.06
Guinea-Bissau	3.93	0.06	82.79
Burundi	2.66	0.03	56.65
Nigeria		0.04	91.85

Source: World development indicators. Data are for 2019. For some SSA countries, data for 2019 are missing, in which case we use data for the latest year available (2017)

## COVID-19 and Digital Infrastructure in Africa

Digital infrastructure includes broadband infrastructure, data infrastructure, and cloud computing infrastructure (Banga, 2017). Digital infrastructure development in Africa has been affected by the COVID-19 pandemic through both supply- and demand-side disruptions.

On the supply side, digital infrastructure development is directly affected by workers falling sick or low-cost telecom operators—usually based in low-income populations in developing countries—exiting the industry due to reductions in consumer spending (ITU, 2020a, 2020b). Indirect supply side-disruptions include closures or suspensions of ICT hardware manufacturing operations due to national lockdowns, as well as shortages of imported materials due to lockdowns and travel bans placed in key trading partners. For instance, the initial lockdown in Wuhan is expected to have a spillover effect on future digital strategies (of 5G technology and fibre optic roll-out plans) of other countries, given that Wuhan is home to Fiberhome, YOFC, and Accelink, among other companies, which together comprise 25% of the global optical fibre production capacity (Cabling, 2020). On the demand side, policies adopted across countries to curtail the spread of the virus—such as lockdowns, travel bans, and work-from-home arrangements—have led to a spike in the demand for internet. Internet traffic has increased by roughly 30% (ITU, 2020a, 2020b). The use of communication apps such as WhatsApp has doubled (USwitch, 2020), and daily usage of some video streaming services has increased by 20 times. Supply-side disruptions to other sectors have also led to an indirect increase in the demand for internet. For instance, the closure of shop outlets and travel bans during the pandemic act as an incentive for non-digitalized firms to switch to online selling to cope with economic losses.

The already existing digital divide between Africa and the rest of the world was not only brought into the spotlight during the COVID-19 but risks being further exacerbated due to countries in Africa witnessing a higher negative supply shock and a lower positive demand shock (Banga & te Velde, 2020). Supply shocks are likely to impact African countries more adversely than developed countries, because of Africa’s lower capacity to substitute imports with domestic manufacturing (IFC, 2020). The increase in internet traffic in African countries is likely to have a supply-side effect of accelerating capital spending for increasing network capacity, leading to a diversion of resources from other non-urgent projects, including 5G investments (ITU, 2020a, 2020b).

Developed countries are likely to benefit more from positive demand shocks, since they are at more advanced stages in terms of digital infrastructure, with more access to fixed network technologies, larger capacity and more robust networks to deal with the surge in demand for digital infrastructure and rising internet traffic as a result of the pandemic (Strusani & Houngbonon, 2020). By comparison, African countries are likely to have witnessed a lower demand shock because almost 86% of economic activities are informal, limiting the enforcement of social distancing in factories and work-from-home arrangements (IFC, 2020). Moreover, the pandemic has shifted the demand from enterprise to residential broadband access, leading to rising demand for access, particularly through fixed broadband (Katz et al., 2020). African countries lag in terms of fixed broadband access: less than 2% of people have access to fixed broadband in Lesotho, Senegal, Malawi, and many other African countries (see Table 2).

**Fig. 1:**
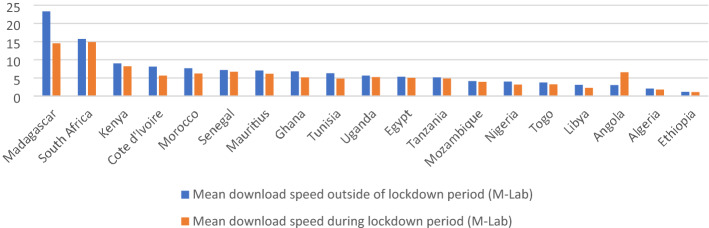
Average internet speeds pre- and post-COVID lockdown in African countries. Source: Authors; constructed from cable.co.uk. Note: Data are based on cable.co.uk’s analysis of the Oxford Coronavirus Government Response Tracker (OxCGRT), and M-Lab’s broadband speed tests. Download speeds are in Mbps

Furthermore, the surge in demand for broadband during the lockdown period^1^ in many African countries has been met through an overall decline in broadband speed (Fig. 1). The three countries with the highest broadband speeds in Africa are Madagascar, South Africa, and Kenya and even these have witnessed a decline of 37.7, 5.4, and 8.5%, respectively, during the pandemic. Reports from the Nigerian Communication Commission suggest that Nigeria gained 2.5 million subscribers during the lockdown in April 2020, with the total number of internet users hitting 143.3 million as of June 2020 (Paul, 2020), although this has come at the cost of a 21% decline in broadband speed (see Fig. 1).

## COVID-19 and Digital Trade in Africa

With the onset of the pandemic, while international trade in goods and services plummeted, digital trade experienced an unprecedented rise. Trade in digital products and digital services via Mode 1 (online) increased exponentially in 2020 and continues to rise. E-commerce has also witnessed exceptional growth. However, the gains from the rise of digital trade have been concentrated in countries with advanced digital infrastructure—i.e., mainly developed countries and a handful of developing countries like China. Africa, as a continent, and most African countries have become net importers of digital goods and services. This section estimates the rise in exports and imports of digital goods and services for Africa.

### COVID-19 and Africa’s Trade in Digital Products

The global demand for digital products—products that are either currently being transmitted through electronic channels or hold the potential to be transmitted electronically in the future—is expected to have risen during the crisis. For instance, the global video game market is forecast to be worth $159 billion in 2020, with close to half (48%) of the industry’s revenue coming from mobile gaming (WEF, 2020). Analysis from GamesIndustry.biz shows that sales across 50 key markets rose by 63%,^2^ with reports from Verizon, a US multinational conglomerate, indicating a 75% increase in gaming traffic in the United States during the quarantine. Reports from Streamlabs indicated a 20% increase in usage hours on platforms such as Twitch, YouTube gaming, and Facebook gaming.^3^ According to Gartner (2021), global IT spending is projected to total $3.9 trillion in 2021—a 6.2% increase from 2020—with a strong rebound in Enterprise Software (8.8% increase) and Data Center software (6.2% increase).

**Fig. 2:**
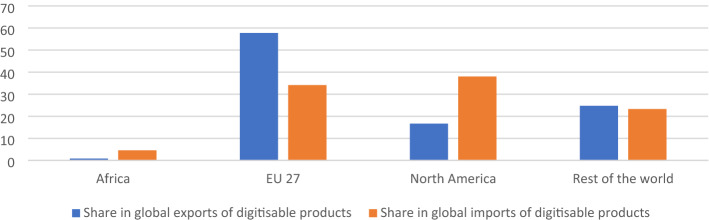
Africa’s share (in %) in global trade of digital products, 2020. Source: Authors; World Integrated Trade Solutions (WITS) dataset. Notes: The graph follows the WTO’s (2016) classification of digital products (or digitizable products), which broadly covers goods such as software, videogames, printed matter, etc. that are either currently being transmitted through electronic channels or hold the potential to be transmitted electronically in the future

Using the WTO’s (2016) classification of digital products (or digitizable products), which broadly covers goods such as software, videogames, and printed matter, Fig. 2 demonstrates that almost 60% of the global exports of digital products in 2020 originated from the EU, with another 16% from North America. Africa’s share in global exports of digital products was almost negligible, at less than 1%. Its share in global imports of digital products is 4.5% (Fig. 2). This has important implications for Africa’s revenue generation, particularly for post-COVID recovery. African countries are net importers of digital products and are unable to tax the imports of these digital products due to a moratorium^4^ on custom duties on electronic transmissions. The potential tariff revenue loss due to this moratorium is estimated to be $ 2.6 billion for African countries, with the tariff loss in SSA estimated to be double that of high-income WTO countries (Banga, 2019). Recent analysis suggests that the potential tariff revenue loss due to the moratorium in the period from 2017 to 2020 exceeded $500 million in Nigeria, South Africa, and Tunisia, with substantial losses of over $100 million estimated for Ethiopia, Malawi, Rwanda, and Zambia (Banga, 2022). The proposal of a permanent moratorium, tabled by several developed countries at the WTO, implies that African countries will be further trading off tariff revenues without even knowing what products will be digitalized in the future. One such example is the gradual rise in electronic SIM cards or e-SIM cards to replace physical SIM cards.

**Fig. 3:**
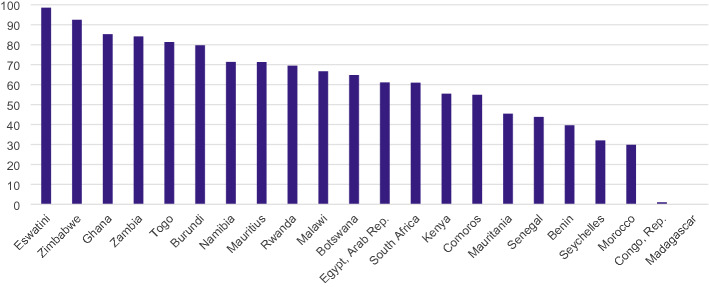
Share of intra-African exports among a country’s total exports of digital products (%). Source: Authors, using the WITS and WTO’s (2016) HS six-digit classification of products that can be electronically transmitted

In terms of exports of digital products, intra-African trade holds important potential, underscoring the importance of the African Continental Free Trade Agreement (AfCFTA) in facilitating exports of digital products for post-COVID recovery in Africa. Bi-lateral trade data analysis (Table 3) for 28 African countries in 2019 revealed that intra-African exports of digital products were valued at $257 million. South Africa, Mozambique, Kenya, Tanzania, and Mauritius emerged as the top five African countries driving intra-African exports of digital products. Over 70% of exports of digital products by Burundi, Eswatini, Ghana, Mauritius, Namibia, Rwanda, Togo, Zambia, and Zimbabwe are intra-African (Fig. 3).

**Table 3:** Intra-African trade in digitizable products, average from 2017–2019

	Share in intra-African imports (%)	Intra-African imports (in $1000 USD)	Share in intra-African exports (%)	Intra-African exports (in $1000 USD)
South Africa	31.60	108,409.39	46.17	124,263.43
Mozambique	3.20	11,773.92	16.30	52,928.47
Kenya	1.44	4788.87	14.90	40,675.42
Tanzania	3.27	12,122.97	11.90	42,577.13
Mauritius	0.40	1317.18	4.30	12,245.14
Eswatini	4.60	14,850.71	3.69	10,406.23
Egypt	0.04	136.95	2.18	5610.38
Uganda	1.86	6442.01	2.17	7204.91
Botswana	4.63	15,091.95	1.79	5226.27
Morocco	1.48	4739.69	1.47	3872.62
Tunisia	0.12	401.20	1.45	3881.18
Ghana	0.69	2304.76	1.08	2725.06
Senegal	0.35	1114.15	0.81	2116.06
Cameroon	1.07	3284.46	0.52	1508.15
Côte d'Ivoire	0.69	2288.70	0.50	1304.01
Zimbabwe	3.77	12,738.38	0.33	882.27
Togo	0.65	2076.58	0.18	471.29
Malawi	15.57	49,621.71	0.14	425.42
Benin	0.28	905.61	0.12	339.84
Nigeria	1.44	4475.56	0.10	368.61
Namibia	11.39	38,260.62	0.10	267.44
Lesotho	2.63	8096.97	0.09	275.88
Zambia	4.98	16,337.27	0.09	256.99
Ethiopia	0.97	3377.55	0.08	252.45
Rwanda	2.46	8003.87	0.04	98.39
Burundi	0.98	3195.41	0.03	95.50
Angola	2.80	11,039.58	0.02	60.91
Burkina Faso	0.34	1100.26	0.02	57.13
Mali	0.16	498.43	0.01	28.32
Madagascar	1.30	4419.12	0.01	27.00
Seychelles	0.83	2753.48	0.00	7.61
Niger	0.10	354.64	0.00	7.88
Sierra Leone	0.23	908.33	0.00	1.79
Comoros	0.01	41.99	0.00	1.28
Republic of Congo	0.08	253.35	0.00	0.97
Sao Tome and Principe	0.01	23.95	0.00	0.50
Mauritania	0.08	246.84	0.00	0.28
Cape Verde	0.01	41.42		
Central African Republic	0.17	625.47		
Gambia	0.07	253.71		

Source: Authors, using the WITS and WTO’s (2016) HS six-digit classification of products that can be electronically transmitted

### COVID-19 and Africa’s Trade in Digitally Deliverable Services (DDSs)

As per the UNCTAD Technical Note (2021), global ICT services’ exports reached $676 billion in 2020, with COVID-19 lockdown restrictions boosting the usage of communications services, computer services, and software. As more businesses in traditional sectors, such as garments, shifted online and consumers worked from home, the share of DDSs among total service exports increased to nearly 64% (UNCTAD, 2021a, 2021b). The rising importance of ICT services was mirrored in Kenya, Africa’s digital leader, with an estimated cloud service market at $275 million (Frost and Sullivan, 2018). In Kenya, the lockdown generated more demand for communications, computers, and information services. Safaricom, for instance, saw a 70% surge in data usage as Kenyans stayed at home to curb the spread of COVID-19 (Reuters, 2020). There was a further boost to the sector through a rise in government demand for ICT services during COVID-19. At all levels—local, national, regional, and continental—African policymakers took to the implementation of digital solutions in their fight against COVID-19, particularly in the case of EdTech and HealthTech. For example, by May 2020, the ministries of education in 27 African countries were providing e-learning platforms for students (UNESCO, 2020). Similarly, the Africa Centres for Disease Control and Prevention launched a continental e-platform to help African governments procure diagnostic tests and medical equipment from certified suppliers on the global market.^5^

There may be new opportunities, particularly in the case of cloud services and data hosting (Deloitte, 2020) for developing countries that have a supportive data and privacy framework. However, more than 75% of the cloud computing market is currently dominated by the United States and China (UNCTAD’s Digital Economy Report, 2019). Access to and usage of cloud- and data-hosting services in emerging African markets also relies on data centres outside local markets, with limited domestic capabilities. Moreover, several African countries do not have broadband speeds that are adequate and affordable enough to support reliable cloud service usage, and these have further declined during the lockdown, as discussed above. This could explain why the public cloud market in Africa is relatively small: less than 1% of estimated global public cloud services revenue was generated in Africa as of 2018, led by South Africa, Mauritius, Kenya, Tunisia, and Morocco (Xalam Analytics, 2018).

So how realistic is a digital services-led recovery for African countries, and can African countries step up to the meet the rise in demand for digital and digitally enabled services? Using data from the OECD-WTO’s Bi-lateral Trade in Services (BaTIS) dataset, Table 4 presents an analysis of African trade in digitally deliverable services (DDSs), defined as an aggregation of ICT services, as well as those services which can be digitally delivered, such as insurance and pension services, financial services, charges for the use of intellectual property, and other business services and audio-visual and related services.^6^ DDS trade in Africa is over $24 billion but it is dominated by a handful of economies: Ghana (25% of Africa’s DDS trade), Morocco (18%), South Africa (15%), Algeria (7%), Kenya (5%), and Nigeria (4%). On average, DDSs form less than 30% of African countries’ services trade, with the share of DDSs among total services being less than 10% in some African countries, such as Tanzania, Ethiopia, Mozambique, Ethiopia, Gambia, and Namibia, but as high as 81% in Ghana. In some African countries, such as Senegal, Niger, Côte d'Ivoire, Zambia, Guinea, and Mauritania, the share of DDSs among total services trade declined after 2015. As such, we should question the potential for a DDS-led post-COVID recovery in these economies. The affordability of digital services and digital devices remains a key barrier in many African countries (Research ICT Africa, 2018).

**Table 4:** African trade in digitally deliverable services

	US dollars, millions	Share of DDS in country's total trade in services	Change in share of DDS trade in 2019 as compared to 2015	Country's share in total Africa's DDS trade
Ghana	6166.10	81.43	4.31	25.40
Morocco	4483.96	23.15	2.43	18.47
South Africa	3791.92	25.75	0.09	15.62
Algeria	1845.12	61.46	− 1.31	7.60
Kenya	1219.31	26.23	4.53	5.02
Nigeria	1068.37	21.59	6.99	4.40
Cameroon	775.92	32.37	3.34	3.20
Mauritius	747.03	25.33	− 7.29	3.08
Senegal	449.15	31.61	− 8.21	1.85
Seychelles	359.60	32.01	3.79	1.48
Tanzania	321.73	8.01	− 4.88	1.33
Uganda	266.61	13.14	− 9.73	1.10
Ethiopia	262.98	5.35	− 0.07	1.08
Côte d'Ivoire	241.96	21.51	− 11.73	1.00
Mali	229.90	38.27	− 9.12	0.95
Botswana	221.10	22.98	1.05	0.91
Togo	209.47	33.68	8.40	0.86
Madagascar	195.08	14.88	− 0.45	0.80
Burkina Faso	190.09	34.12	2.52	0.78
Sudan	145.37	10.63	6.38	0.60
Benin	132.11	26.25	11.97	0.54
Zambia	114.39	11.28	− 2.90	0.47
Malawi	101.15	56.51	11.26	0.42
Niger	98.64	39.94	− 15.17	0.41
Libya	82.10	61.41	− 31.76	0.34
Mozambique	72.09	7.81	− 5.12	0.30
Guinea	59.39	57.11	− 27.77	0.24
Mauritania	57.91	34.73	− 23.04	0.24
Rwanda	57.34	6.25	− 1.16	0.24
Zimbabwe	55.14	13.16	− 6.61	0.23
Sierra Leone	47.17	40.57	− 18.06	0.19
Angola	42.65	7.63	2.22	0.18
Eswatini	39.64	44.64	17.64	0.16
Namibia	38.58	5.93	− 2.58	0.16
Comoros	21.95	20.24	− 4.99	0.09
Burundi	16.82	16.03	− 9.32	0.07
Gambia	10.54	4.60	2.52	0.04
Liberia	10.14	13.91	11.53	0.04
D.R. Congo	9.84	8.57	− 4.56	0.04

Source: Authors, constructed from OECD-WTO’s Balanced Trade in Services (BaTis) dataset

Note: Data are for 2019 or for the latest year available

**Fig. 4:**
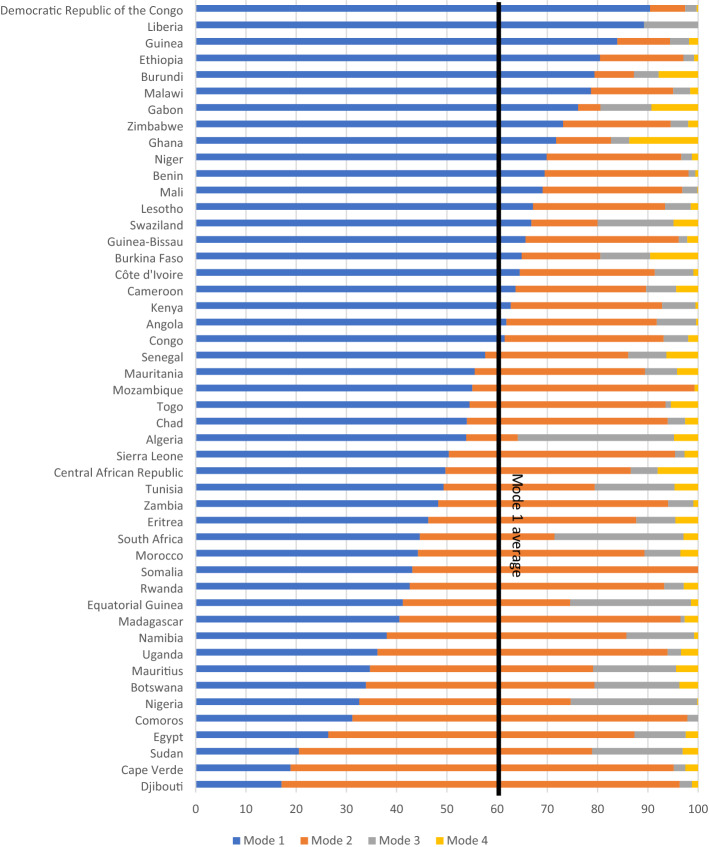
Supply of services in African countries, by mode of supply. Source: Authors, calculated using OECD-WTO’s TisMOS database. Note: Mode 1 (services provided by phone, fax, or electronic means), Mode 2 (people travelling to another country to consumer services locally), Mode 3 (commercial presence), and Mode 5 (presence of natural persons)

Examining the current modes of cross-border supply in services trade of African countries (Fig. 4), we note that in some countries, such as D.R. Congo, Liberia, Ethiopia, Ghana, Burundi, and Malawi, more than 60% of services are being supplied online (Mode 1). But this falls to less than 40% in in Uganda, Botswana, Nigeria, Comoros, Mauritius, Namibia, Egypt, and Sudan. This implies that the supply of services in the latter set of countries was more significantly disrupted during the pandemic. In any case, even in the countries where the majority of services are supplied through Mode 1, resilience to economic shocks, such as the pandemic, is contingent on good and reliable access to the internet as well as appropriate ICT regulatory frameworks.

### COVID-19 and African e-Commerce

COVID-19 has given a boost to e-commerce globally. Physical distancing, national lockdowns, and other containment measures developed by various countries to curtail the spread of the virus have generated a direct positive demand shock to both business-to-business (B2B) and business-to-consumer (B2C) online sales, particularly in medical supplies, household essentials, and food products (WTO, 2020). The African platform Jumia, for instance, saw a 50% increase in the volume of transactions during the first six months of 2020, compared with the same before (UNCTAD, 2021b). The closure and suspension of manufacturing operations and shop outlets, as well as travels bans, also led manufacturing firms to increase their share of online sales.

**Fig. 5:**
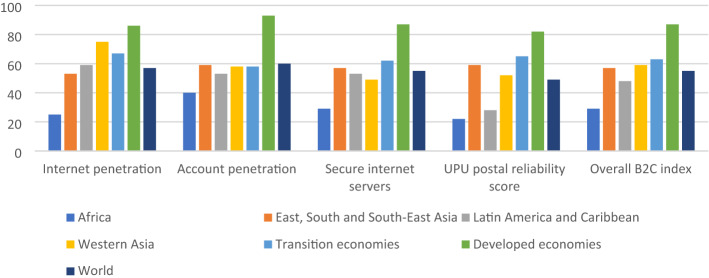
B2C e-commerce index, 2019. Source: UNCTAD B2C e-commerce index, 2019

But the accelerating impact of COVID-19 on e-commerce in Africa has been overall constrained by persisting weaknesses in the continent’s digital economy that continue to frustrate e-commerce development (Futi AND Macleod, 2021). As noted by UNCTAD (2021a, 2021b), the Middle East and Africa together accounted for less than 3% of global e-commerce sales in 2020. Moreover, within Africa, just 10 countries are responsible for 94% of all online business on the continent (ITC, 2020). Africa ranks the lowest on the B2C e-commerce index developed by UNCTAD, with a value of 29 as compared to 87 in developed countries and 55 as the world average (Fig. 5). This index draws information on internet and account penetration, internet servers, and postal reliability, and finds that Africa particularly lags in the case of postal reliability. Furthermore, 57% of marketplaces allow only domestic sellers on their platform and only 28% of those operating in Africa offer online payment transactions, which further restricts cross-border e-commerce (ibid.). Other challenges to African e-commerce include high internet costs and cross-border trade costs (Futi & Macleod, 2021), in addition to issues of taxation (e.g. double taxation and VAT regulations), unawareness of national and regional rules, custom duties and custom procedures, a lack of digital trust, and poor online consumer protection (Banga et al., 2021). There have also been supply-side disruptions to African e-commerce during COVID-19 in the form of travel disruptions, delays in parcel delivery due to cargo, air, and transport disruptions, increasing air freight prices, a shortage of qualified workers, and poor delivery infrastructure. The analogue and digital challenges related to e-commerce underscore the important role that AfCFTA can play in developing an effective e-commerce protocol that is coherent with other protocols, such as Trade in Goods, to boost e-commerce on the continent.

## Digital Responses to COVID-19 and Economic Performance of African Firms

More than one in five firms in SSA started or expanded their use of digital technology in response to the COVID-19 shock, but some regional differences have been noted in the digital response to COVID-19 in Africa (World Bank, 2021). Firms in East and Southern Africa were more likely than those in West and Central Africa to have started or expanded their use of digital platforms, with 27 and 20% of firms doing so, respectively (World Bank, 2021). However, the propensity to use digital platforms on the continent remains smaller than that of firms in other developing countries (32%), with the conditional likelihood of having initiated or expanded the use of digital platforms being less than 10% among firms in Niger, Chad, and Tanzania (Davies et al., 2021).

Using the World Bank’s (2021) Impact of COVID survey Round 1 for the year 2020, we undertook an analysis to examine whether firms with a digital response to COVID-19 fared better in terms of economic performance compared to firms that did not adopt a digital response. Table 5 shows that of 2383 firms (in manufacturing, retailer services, and other services) across selected African countries, only 538 firms (22.5%) adopted a digital response to the pandemic in 2020, i.e. reported starting or increasing online business activity. Of 927 manufacturing firms, 219 firms (23.6%) adopted a digital response.

**Table 5:** African firms with a digital response to the pandemic

	Number of firms with a digital response to COVID	Number of firms interviewed	% of firms with a digital response to COVID	Number of manufacturing firms with a digital response to COVID	Number of manufacturing firms interviewed	% of manufacturing firms with a digital response to COVID
Chad	6	101.00	5.94	0	54.00	0.00
Guinea	21	103.00	20.39	1	17.00	5.88
Morocco	216	778.00	27.76	105	326.00	32.21
Mozambique	29	219.00	13.24	10	86.00	11.63
Niger	5	65.00	7.69	2	18.00	11.11
Togo	6	49.00	12.24	1	15.00	6.67
Zambia	130	532.00	24.44	33	147.00	22.45
Zimbabwe	125	536.00	23.32	67	264.00	25.38
Total	538	2383		219	927	

Source: Authors, using data from the World Bank’s (2021) Impact of COVID survey

Table 6 focuses on the 927 manufacturing firms and examines the percentage of firms impacted by supply- and demand-side disruptions due to COVID-19 across their digital responses. It is observed that a higher share of firms *without* a digital response to the pandemic suffered negative demand and supply shocks as compared to firms with a digital response. Firms with a digital response were more likely to have higher resilience to the pandemic compared to firms without a digital response.

**Table 6:** Percentage of firms impacted by supply- and demand-side disruptions across digital responses

	No digital response to COVID-19	Digital response to COVID-19
Negative demand shock due to COVID-19		
Decrease in demand	82.41	76.08
Decrease in monthly sales	87.64	83.11
Decrease in monthly exports	42.60	43.90
Negative supply shock due to COVID-19		
Decrease in total hours worked per week	69.31	56.16
Decrease in supply of inputs, raw materials, or finished goods and materials purchased to resell	80.00	76.20
Positive demand shock due to COVID-19		
Increase in demand	4.12	7.66
Increase in monthly sales	2.98	5.02
Increase in monthly exports	0.48	3.38
Positive supply shock due to COVID-19		
Increase in total hours worked per week	1.70	0.91
Increase in supply of inputs, raw materials, or finished goods and materials purchased to resell	4.54	5.94
Resilience to COVID-19		
No change in monthly sales	9.38	11.87
No change in monthly exports	10.33	13.53
Adjusted or converted, partially or fully, its production or the services	38.33	63.01
No change in total hours worked per week	29.00	42.92
No change in demand	13.47	16.27
No change in supply of inputs, raw materials, or finished goods and materials purchased to resell	15.46	17.81

Source: Authors, using data from the World Bank’s (2021) Impact of COVID survey

**Fig. 6:**
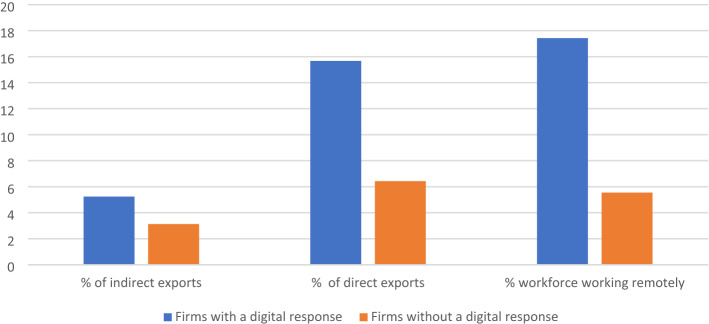
Growth outcomes across digital responses to COVID. Source: Authors, using data from the World Bank’s (2021) Impact of COVID survey. Note: Differences are significant at 5%

Further, we conducted *t*-tests to examine whether export performance differed significantly between firms with and without a digital response to the pandemic. Figure 6 shows that African manufacturing firms with a digital response reported a significantly higher share of sales exported directly or indirectly, and a higher share of the workforce working remotely. Differences are significant at 5%.

## National Digital Industrialization Policies: Lessons from Other Developing Countries

In the light of the analysis above, Africa was found to lag in terms of its digital trade in products, services, and e-commerce. This digital divide has adversely impacted Africa’s opportunities to digitally trade during the pandemic. It is therefore important to design comprehensive national digital policies in African countries, supported by regional digital cooperation and facilitated by international cooperation on digital transformation. A study of 60 policies in SSA suggests that post-COVID-19, the region is focusing on areas of ICT affordability (44%) and network expansion (28%), with South Africa implementing the largest number of measures, followed by Ghana (World Bank, 2021). Beyond targeting ICT access, African countries need to design comprehensive national digital industrialization policies that emphasize investments in not only broadband and ICT infrastructure but also data infrastructure and national e-commerce platforms. Such policies can boost the digital content in the production and supply of industrial products, leading to higher African trade competitiveness (UNCTAD, 2018).

Data are at the heart of digital industrialization. It is often said that data are the new oil, although few understand the analogy. During the first industrial revolution, it was not the oil producing countries but rather the oil processing countries that developed. Similarly, in digital revolution, it is the data processing countries that have a higher potential to digitally transform. With the world’s highest young population, and therefore a data mine for the world, it is critical that African countries develop their data processing capacities to reap gains from the data they produce every second. For developing data processing capacities, African countries need to put in place appropriate data regulation policies that declare their sovereign rights over data. This will allow them to build their data infrastructure and digital skills.

China is a successful example of a developing country that has been able to transform itself from an agrarian economy to a global manufacturing hub and then to an emerging digital leader. There has been a big push in China for the development of network infrastructure, including cloud computing infrastructure and data analytics, and sector-specific digital transformation policies, as well as data governance and data regulation policies (Wei and Xin, 2020). China’s Cyber Security Law recognizes the importance of data ownership and data sovereignty. It stipulates that the personal information and important data collected and generated in domestic operations of critical information infrastructure operators shall be stored within China's territory, and where such data are transferred across borders for business needs, security assessments shall be conducted. In Sri Lanka, the urgent need for data protection has led to preparation of a draft bill, under which a high-level task force is required to establish a data protection authority (DPA) within a specified period. As noted by Wattegama (2021), several obligations have been imposed by this legislation on those who collect and process personal data (“controllers” and “processors”), and a whole new set of rights have been given to citizens under this new legislation, known as the “rights of data subjects”. For instance, personal data can be collected only for a specified purpose and must be processed in a manner to ensure appropriate security, including protection against accidental loss, destruction, or damage. In Indonesia, under the Personal Data Protection No. 20 of 2016, an individual has the following rights to their data: access and update, deletion, destruction, and the right to be forgotten (Esther, 2021).

African countries also need to develop their national e-commerce platforms to link their small and medium enterprises to international markets. However, it needs to be noted that e-commerce platforms are two-sided platforms. On one hand, they can enable domestic producers to access global markets. But on the other hand, they also provide access to domestic markets for global exporters. Regulating imports through e-commerce platforms may be a useful policy to consider for African countries. It may also be important to regulate foreign e-commerce platforms and put in place strong competition policies so that the foreign platforms do not out-compete national infant e-commerce platforms in African countries. Only 28 African countries currently have competition authorities to investigate and address anticompetitive behaviour. Even in the countries that have such authorities, there are issues such as a limited capacity to regulate (Futi & Macleod, 2021). Regulating the operations of e-commerce platforms is also needed. For example, India has recently announced its national FDI e-commerce policy, which will help to promote fair competition in India’s fast-growing domestic e-commerce market.

China, for instance, has detailed cross-border e-commerce policies to promote exports of its e-commerce platforms and regulate imports through e-commerce platforms. It encourages e-commerce platforms that sell only Chinese products, such as KIKUU, which operates in more than six African countries. On imports through e-commerce platforms, China follows a positive list approach. On March 17, 2017, the Ministry of Commerce made it clear that cross-border e-commerce retail imports will be regulated in terms of personal items. That is, not everything available can be imported through the e-commerce platforms (Chen, 2020).

## Digital Cooperation at Regional Level for Digital Transformation of Africa

### Regional Digital Cooperation Agenda

At the regional and sub-regional levels, digital cooperation can help African countries to digitally develop and progress rapidly towards achieving transformational growth. UNCTAD proposed a 10-point Regional Digital Cooperation Agenda for industrialization and regional integration,^7^ which outlines the ways in which regional economic communities (RECs) like COMESA, EAC, etc. can progressively digitalize, boosting their digital industrialization as well as strengthening regional integration. This involves building a regional data economy, building regional cloud computing infrastructure, strengthening regional broadband infrastructure, promoting regional e-commerce, promoting regional digital payments, progressing towards a single digital regional market, sharing experiences on e-government, forging regional partnerships for building smart cities, promoting regional innovations and technologies, and building regional statistics for measuring the extent of digitization.

South–south cooperation in these areas, especially at the regional level, can provide an important way forward for African countries. Small African countries will need regional support to develop their digital and financial infrastructures to use digital technologies. Anti-competitive strategies can be designed at the regional level to protect their regional and national e-commerce platforms and boost fair competition. Given economies of scale in digital infrastructure such as cloud computing infrastructure, it will be much more advantageous for African countries to cooperate to build this infrastructure.

It is also important for African countries to be cognizant of the interdependencies of their growth in the emerging digitalized global economy. Big developing countries have the potential to act as digital growth pole for other smaller and low-income developing countries. This in turn will further boost growth in these big developing countries, given the scale economies of digital technologies. But this win–win option available to the Africa can be delivered only through stronger south–south cooperation. Policymakers should recognize the potential of south–south cooperation in the digital world and design national policies that can help this cooperation to progress towards productive integration.

### Regional Cooperation to Build Smart Societies

African countries face similar challenges when it comes to building smart societies, which go hand in hand with building smart cities. At the regional level, targeted policies need to be added to already existing regional integration agendas for building smart societies in Africa. Countries will need regional support in order to provide smart governance and smart social services in the areas of health, education, urbanization, and disaster management. Developing public e-services using digital technologies and sharing them within the region can be economically more sustainable than countries developing their own smart governance services. Digital start-ups can be encouraged to develop e-governance services for Africa that can benefit all countries of the regional blocs and the continent.

### Role of Regional Development Banks

Regional development banks like African EXIM Bank and Africa Development Bank can play an important role in digitally advancing their member countries in Africa. A digital development fund can be set up at the regional level to provide support to digital initiatives at the national level. Countries can contribute to this fund, which can then finance digital initiatives at the regional level. Further, to recover faster from COVID-19 and to recover better with resilient growth, African countries will need financial support. Regional development banks need to be given additional capital injections so that they can help African countries revive their economies post-pandemic. Small and medium-sized firms will require subsidized loans, subsidized inputs, and adequate infrastructure support for them to survive the pandemic and rebalance their growth. While developed countries are rolling out billions of dollars in recovery packages, African countries may not have the financial leverage to provide economic stimulus packages and would have to work with their SMEs through other channels of support using regional development banks.

## International Cooperation for Digital Transformation

The fourth digital revolution has brought new opportunities for transformational growth for Africa. But these opportunities have also ushered in formidable challenges. The global community will need to work closely in order to bridge the digital divide and help African countries recover from the pandemic with resilient growth and achieve digital transformation. International cooperation is needed in order to use digital technologies and digital services to help countries progress in their SDGs. An SDG fund can be an important source for channelling resources for building digital capacities in southern countries, especially in Africa. To rebuild Africa’s trade competitiveness in the digital world, it is important that international bodies like the WTO provide adequate policy and fiscal support to African countries. Some of the ways in which this can be done are discussed in what follows.

### Removal of WTO e-Commerce Moratorium

As more and more products digitalize in the digital era, the removal of the WTO e-commerce moratorium can provide a continually growing source of tariff revenue to African countries, especially given that most African countries are net importers of electronic transmissions (Banga, 2019). For instance, the number of subscription video-on-demand users in Africa is expected to reach a sizeable 15 million by 2026, with Netflix expected to have the highest number of subscribers.^8^ Retaining policy space to tax luxury online imports, such as movies and videogames, can therefore be an importance source of additional revenue generation for post-COVID recovery.

### Re-invigorating the Work Program on e-Commerce

Instead of focusing on how to deliver gains from growing global e-commerce to developing countries and building their digital capacities for increasing their exports, as mandated by the Doha Development Agenda, some countries are negotiating digital rules under the Joint Initiative Statement on E-Commerce, which is fracturing the multilateral process and diverting attention from the e-commerce work program instituted within the WTO. These digital rules can have high compliance costs for African countries, and they can limit their policy space for designing digital industrialization policies.

WTO members need to reinvigorate the e-commerce work program by focusing on building awareness of the members on the development implications of growing global e-commerce, and by finding ways to increase the export competitiveness of their SMEs. The Africa group in the WTO can greatly help by supporting this proposal at the WTO.

### Cooperation on Financing Digital Transformation in Africa

Important challenges to digital infrastructure development in Africa include the high costs of financing digital technologies and poor access to credit. This has been an important reason for under-investment in the digital transformation of Kenyan manufacturing industries, particularly the textile and apparel industries, where the cost of financing manufacturing investments and trade financing is already very high compared to global rates (KAM, 2018). Recently, securing loans from SMEs through development finance institutions (DFIs) lending to banks has been picking up momentum. For instance, in 2018, the Kenyan I&M Bank secured a $40 million loan from the Dutch Development Bank (FMO) for onward lending to SMEs (Business Daily, 2018). African countries can also liaise with institutions such as the African Development Bank (AfDB), which can provide support to local banks through partial guarantee facilities, as well as the World Bank, which can directly finance an enabling environment for manufacturing.

## Conclusions and Ways Forward

This paper highlighted the existing global digital divide (with Africa lagging far behind other developing regions) as well as the intra-Africa digital divide (with some countries lagging substantially behind others in terms of even internet access). We also examined the demand- and supply-side effects of the COVID-19 pandemic on Africa’s digital infrastructure, which has in turn impacted Africa’s digital trade.

In 2020, Africa’s share in global exports of digital products was less than 1%, while its share in global imports of digital products was 4.5%. On average, the share of digitally deliverable services (DDS) in the service sector in Africa is less than 30%, and has in fact declined since 2015 in Senegal, Niger, Côte d'Ivoire, Zambia, Guinea, and Mauritania. This should lead us to question the potential for a DDS-led post-COVID recovery. Africa’s share of global e-commerce has also remained low, due to poor internet access, low broadband speeds, and few national e-commerce platforms. Very few firms in Africa were able to offer a digital response to the pandemic. As such, Africa has lost opportunities to gain from the rise in digital trade.

A lack of comprehensive policies for digital transformation at the continental, regional, and national levels was found to be one of the main reasons for Africa’s sluggish growth in digital trade. In what follows, we propose ways forward for African countries in the digital era and suggest policies which can be designed at all levels for a digital transformation of Africa. At the national level, drawing from China’s successful digital transformation experience, African countries should design comprehensive national digital policies. These policies should give a big push to developing digital infrastructure, including ICT infrastructure, broadband infrastructure, data infrastructure, and cloud computing infrastructure. Digital industrialization policies need to be designed by keeping data at the core and aiming to develop digital skills in labour and big data analytical capacities for firms. For this, ownership of data, along with declaring sovereign rights over national data, is extremely important. African countries need to develop and promote national e-commerce platforms. Strong competition policy is needed in order to protect national platforms from undue competition from foreign digital super-platforms.

At the regional level, African countries should adopt the 10-point regional digital cooperation agenda proposed by UNCTAD (2018) to boost regional integration and industrialization. Small African countries will need regional support to develop digital infrastructure and provide the much-needed scale economies to small African firms. At the continental level, AfCFTA can play an important role in facilitating Africa’s digital transformation and exports of digital products for post-COVID recovery. Over 70% of exports of digital products by Burundi, Eswatini, Ghana, Mauritius, Namibia, Rwanda, Togo, Zambia, and Zimbabwe were intra-African. AfFCTA needs to promote digital cooperation frameworks. It is also important that Africa supports the removal of the WTO e-commerce moratorium to be able to regulate imports of digital products—that is, luxury items such as video games, movies, and music—especially in the COVID era, not only to raise additional revenue for governments but also to preserve domestic financial resources.

## Data Availability

Data available on request.

## Abbreviations

DDS: Digitally deliverable service
UNCTAD: United Nations Conference on Trade and Development
IATA: International Air Transportation Association
UN: United Nations
UNWTO: United Nations World Tourism Organisation
WTO: World Trade Organisation
GDP: Gross domestic product
ITU: International Telecommunication Union
OxCGRT: Oxford Coronavirus Government Response Tracker
ICT: Information and Communication Technologies
BaTIS: Balanced trade in services
AfCFTA: African Continental Free Trade Agreement
TisMOS: Trade in services mode of supply
B2C: Business to consumer
EXIM: Export–import
COMESA: Common Market for Eastern and Southern Africa
EAC: East African Community

## Footnotes

1 The lockdown period in a country is identified as the period between the first day on which the Oxford COVID-19 Government Response Tracker stringency exceeded 50 and the last day it exceeded 50. See https://www.bsg.ox.ac.uk/research/research-projects/coronavirus-government-response-tracker for more information on the tracker.

2
https://www.gamesindustry.biz/articles/2020-03-28-what-is-happening-with-video-game-sales-during-coronavirus

3 https://blog.streamlabs.com/streamlabs-stream-hatchet-q1-2020-live-streaming-industry-report-9630bc3e0e1e

4 In 1998, on the basis of a proposal submitted by the United States, WTO members adopted a declaration on global electronic commerce, which included a two-year moratorium or ban on custom cuties on electronic transmissions.

5 https://www.virgin.com/virgin-unite/latest/african-union-launches-ground-breaking-medical-supply-platform-stop-covid-19.

6 The digitally deliverable services series is based on the concept of potentially ICT-enabled services, as developed by UNCTAD in a technical note in 2015, as well as in a report by the 47th United Nations Statistical Commission in 2016.

7 Banga and Kozul-Wright (2018).

8 https://qz.com/africa/2052540/african-streaming-platforms-have-an-edge-over-netflix-and-disney/.
